# RARG重排急性髓系白血病研究进展

**DOI:** 10.3760/cma.j.issn.0253-2727.2022.07.016

**Published:** 2022-07

**Authors:** 庭 史, 鸿鹄 主

**Affiliations:** 浙江大学医学院附属第一医院血液科，浙江大学癌症中心，浙江省血液肿瘤（诊治）重点实验室，浙江省血液病临床医学研究中心，杭州 310003 Department of Hematology, the First Affiliated Hospital, College of Medicine, Zhejiang University, Hangzhou, 310003, China

急性髓系白血病（AML）是一类常见的血液系统恶性肿瘤，约占成人急性白血病的80％，现有治疗手段主要包括化疗和造血干细胞移植（HSCT）。美国SEER数据库最新数据显示AML患者的5年存活率仅为29.5％[1]，因此AML的精准诊疗成为国际研究的重点。

AML是一组高度异质性的疾病[2]。欧洲白血病网络（ELN）和美国国立综合癌症网络（NCCN）指南根据分子遗传学结果，将AML分为预后良好、中等、不良三组，以此为基础的分子分型诊疗策略改善了部分患者的预后[3]。随着二代测序技术的出现和快速应用，发现了越来越多的分子异常，进而认识了更多的AML亚型；迄今为止，根据最新第4版《WHO造血和淋巴组织肿瘤分类》，已定义了19种AML亚型[4]。

然而，WHO分型并非解决了所有诊断分型问题，它是一个开放的系统，有待新的类型被发现并补充。近年来，我们发现一类特殊AML患者，其临床表现、出凝血检查、骨髓细胞形态学、免疫分型与急性早幼粒细胞白血病（APL）非常相似，而分子遗传学检测无t（15;17）/PML-RARA和其他RARA重排，且其对全反式维甲酸（ATRA）和亚砷酸（ATO）治疗亦无效；这类患者在WHO分型中没有描述而无法分型，临床医师通常笼统诊断为AML而进行治疗。后来通过RNA-seq方法，才发现这类患者存在维甲酸γ受体（RARG）和其他基因发生融合；并且随着RNA-seq方法推广和检测费用的下降，越来越多的病例被发现，我们暂定义该类白血病为RARG重排AML（RARG-AML）。本文就RARG-AML的最新认识进行阐述。

一、对RARG-AML疾病的认识过程

2011年西班牙的Sanz教授团队首次报道了1例NUP98-RARG患者[5]，该病例临床、骨髓细胞形态学、免疫分型均具有APL的典型表现，但是无t（15;17）和PML-RARA，染色体发现存在t（11;12）（p15;q13），通过比较基因组杂交（CGH）分析技术最终确定了12q13的RARG基因和11p15的NUP98基因发生融合，这是全球报道的第1例RARG-AML患者。

2017年韩国Ha教授团队报道了1例PML-RARG患者[6]，该患者存在t（12;15）（q13;q22），通过全基因组测序（WGS）分析确定了12q13的RARG基因和15 q22的PML基因发生融合，这是全球报道的第2例RARG-AML患者。

2018年，陈苏宁教授团队、主鸿鹄教授团队和美国华盛顿大学Welch 教授团队同期报道了4例CPSF6-RARG患者[7]–[9]，这些患者的CPSF6-RARG融合基因均由RNA-seq测序发现。从2019年到2021年，又新增了9例RARG-AML的病例报道[10]–[18]，均来自中国学者，新发现了HNRNPC-RARG和NPM1-RARG两种融合基因。截至2022年3月，国际上共有14篇文献报道了15例RARG-AML[5]–[18]，共有5种RARG融合基因类型（NUP98-RARG、PML-RARG、CPSF6-RARG、HNRNPC-RARG和NPM1-RARG）。

2019年2月，主鸿鹄教授在国际上首次提出RARG-AML是一类新的疾病类型[10]，并对该类疾病进行了探索性研究，且于2021年牵头组织了全球RARG-AML协作组，共有37个中心团队参加，包括美国MD. Anderson癌症中心Kantarjian教授（白血病中心主任）团队、美国华盛顿大学Welch教授（TCGA-AML项目领导者之一）团队、西班牙Sanz教授（国际公认的APL危险积分Sanz体系的创建者）团队，以及英国和韩国团队等，希望通过对全球多中心的临床数据和组学数据的深入研究，促进对该类疾病的认识，在国际上建立一个由我国学者首次命名的全新AML类型。

二、RARG-AML的疾病特征

荟萃已发表文献中15例患者的数据[5]–[18]，对RARG-AML的临床特征总结如下：发病中位年龄45（13～67）岁，男9例，女6例。初诊时最常见临床表现为出血（皮肤、黏膜）（50％）、发热（50％）、乏力（36％）；血常规中WBC、HGB、PLT中位值分别为5.3（0.2～112.6）×10^9^/L、80（42～125）g/L、60（18～204）×10^9^/L，WBC<10×10^9^/L的患者占67％；凝血酶原时间（PT）、活化的部分酶原时间（APTT）、纤维蛋白原（Fib）、D-D二聚体的中位值分别为 13.7 s、31.55 s、12.9 mg/L、5 680 µg/L；骨髓形态学诊断APL为100％，其中粗颗粒型占60％，细颗粒型占40％；骨髓免疫分型：CD34^−^（86％）、CD38^−^（100％）、HLA-DR^−^（80％）、CD11b^−^（64％）、CD117^+^（86％）、CD13^+^（93％）、CD33^+^（100％）、MPO^+^（100％）；骨髓细胞遗传学：t（11;12）（p15;q13）4例、正常核型6例、其他异常5例；骨髓分子生物学：CPSF6-RARG 7例、NUP98-RARG 5例、HNRNPC-RARG 1例、PML-RARA 1例、NPM1-RARG 1例。临床应用ATRA和ATO治疗2周以上可评估疗效者中100％无效。1个疗程、2个疗程化疗（“3+7”方案）缓解率分别为33％和67％，早期死亡率为20％。2年复发率64％，2年总体生存率31％。

三、RARG-AML和APL的鉴别

从现有的数据看，RARG-AML和APL从临床表现和常规检查角度难以鉴别。RARG-AML患者临床表现也是以出凝血异常为主并伴有原发性纤溶亢进，两系或三系减少常见，骨髓细胞形态学可见以颗粒增多的异常早幼粒细胞为主并且POX染色强阳性，免疫分型表现为典型APL的特征，即CD34^−^、HLA-DR^−^、CD11b^−^、CD117^+^、CD13^+^、CD33^+^、MPO^+^。从快速诊断角度，FISH方法具有重要的意义；如果拟诊APL患者未检测到17号染色体异常，基本上可以排除经典和变异型APL，需要及时启动RNA-Seq测序。但是由于目前RNA-Seq测序主要为科研服务，检测费用相对较高，检测结果回报时间较长（2周以上），限制了其临床推广应用，而导致多数RARG-AML病例被漏诊或误诊。从已经发表的RARG-AML病例看，从初诊到明确诊断的时间平均50 d（19 d～32个月）。因此亟需开发一套快速多重PCR试剂盒，将5种RARG融合基因进行快速筛查。

四、RARG-AML的治疗

由于RARG-AML不能得到早期诊断，多数患者接受ATRA/ATO治疗1周以上，从而延迟化疗导致疾病进展，容易出现早期死亡，因此需要尽快排除APL的诊断，及时进行“3+7”为主的标准化疗。但是现有的资料显示RARG-AML传统化疗缓解率低，标准化疗诱发出血加重容易发生早期死亡，因此在化疗的同时一定要加强输血支持治疗，保证PLT在30×10^9^/L以上、Fib水平在1500 mg/L以上。目前难以明确最佳的诱导化疗方案，但是少数病例显示出高三尖杉酯碱的高度敏感性，值得进一步探索其疗效以及机制。鉴于RARG-AML化疗后2年复发率高达64％，建议缓解后尽早进行异基因造血干细胞移植。

五、RARG-AML的基础研究

鉴于RARG-AML是一类新的疾病，深入研究其发病机制以及潜在的治疗靶点非常必要。而截止到目前仅有一篇文献对RARG-AML的发病机制进行了初步探讨。2015年美国贝勒医学院Dong教授团队对NUP98-RARG的功能进行了初步研究[19]，利用NUP98-RARG转染Hela细胞，发现该融合蛋白异常核定位，具有和PML-RARA相似的转录活性，NUP98-RARG和维甲酸X受体（RXR）可以形成异源二聚体；小鼠骨髓正常干祖细胞转染NUP98-RARG后获得克隆增殖能力，而RARG的DNA binding domain（DBD）和NUP98的GLFG domain是关键区，NUP98-RARG转化的骨髓干祖细胞对ATRA敏感，处理后分化明显。该研究初步显示了NUP98-RARG的表型，但缺乏系统性的针对多种RARG融合基因的功能和靶点挖掘研究，并且该体外研究的结果和临床ATRA治疗无效的现象矛盾，因此需要更好的模型系统性深入完善研究，这也是国际该领域亟待解决的科学问题。目前我国有4～5个课题组正在进行该领域的基础研究。希望通过全球合作，提升对这类新的AML类型的基础和临床认识水平，建立该病的诊断、分型、治疗标准，从而改善该类疾病的预后。

六、展望

RARG-AML是一类新的白血病类型，具有如下临床和实验室特征：出凝血异常显著、二系或三系减少、骨髓细胞形态学类似APL、免疫分型CD34^−^HLA-DR^−^CD11b^−^CD117^+^CD13^+^、X-RARG融合基因、ATRA/ATO无效、早期死亡率高、生存差。对于临床拟诊APL但是FISH/PCR方法排除APL者应想到RARG-AML的可能性，及时进行PCR检测有利于快速诊断、RNA-seq方法有助于寻找新的RARG融合基因以及其他合并分子异常；在加强输血治疗基础上尽早进行“3+7”为主的联合化疗，缓解后推荐异基因造血干细胞移植，上述策略有助于提高该病的确诊率及治疗反应，降低早期死亡率和复发率，提高生存率。而加强基础研究，阐明该病的发病机制、寻找有效的靶向性药物，建立靶向性药物治疗为主的新方案是未来发展的趋势。
